# Antibiotic Resistance: What are the Opportunities for Primary Care in Alleviating the Crisis?

**DOI:** 10.3389/fpubh.2015.00035

**Published:** 2015-02-24

**Authors:** Malene Plejdrup Hansen, Tammy C. Hoffmann, Amanda R. McCullough, Mieke L. van Driel, Chris B. Del Mar

**Affiliations:** ^1^Centre for Research in Evidence-Based Practice, Bond University, Gold Coast, QLD, Australia; ^2^Discipline of General Practice, School of Medicine, University of Queensland, Brisbane, QLD, Australia

**Keywords:** antibiotic resistance, primary care, prescriptions, behavior, probiotic, steroids

## Abstract

Numerous opportunities are available in primary care for alleviating the crisis of increasing antibiotic resistance. Preventing patients from developing an acute respiratory infection (ARI) will obviate any need for antibiotic use downstream. Hygiene measures such as physical barriers and hand hygiene, and possibly vaccination and exercise, may be effective. Also, a large range of complementary and alternative medicines (e.g. zinc, vitamin C and probiotics) are proposed for preventing and treating ARIs, but evidence for efficacy is scarce.

General practitioners’ (GPs) attitudes towards antibiotic prescribing are a major factor in the prescribing for ARIs. Professional interventions with educational components are effective, although they have modest effects, and are expensive. GPs’ perceptions – that mistakenly assume as a default that patients want antibiotics for their ARIs – are often wrong. Shared decision making might be a solution, as it enables clinician and patient to participate jointly in making a health decision, having discussed the options together with the evidence for their harms as well as benefits.

Furthermore, GPs’ diagnostic uncertainty – often leading to an antibiotic prescription “just in case” – might be addressed by exploiting strategies such as safety-netting, e.g., establishing with the patient *a priori* clearly defined actions to take if the course of the illness deviates from the expected.

None of these strategies or interventions on their own will greatly improve the use of antibiotics for ARIs. However, used in concert, combinations are likely to enable clinicians and health care systems to implement the strategies that will reduce antimicrobial resistance in the future.

## Setting the Scene

In the past 70 years, antibiotics have been essential in the fight against infectious diseases and have been a contributing factor in the rise in life expectancy (1). However, we are now gradually facing a post-antibiotic era, a time when antibiotics are no longer effective because bacteria have become more and more resistant. The World Health Organisations’ 2014 report on global surveillance of antimicrobial resistance reveals that antibiotic resistance is no longer a prediction for the future; it is happening right now, across the world, and is putting at risk the ability to treat common infections in both the community and hospitals (2). Excessive and inappropriate use of antibiotics is considered to be the most important cause of the increasing problems with resistant bacteria (3). Consequences are not only at a population level but also for individual patients, as it is shown that individuals who are prescribed an antibiotic for a respiratory or urinary infection can develop bacterial resistance to that antibiotic for up to 12 months (4).

The majority of antibiotics are prescribed in primary care (5) and mainly for acute respiratory infections (ARIs) (6). ARI is an overall term for a group of illnesses and the most common infections presenting in primary care are acute otitis media, acute sinusitis, acute tonsillitis, acute pharyngitis, acute bronchitis, pneumonia, the common cold, and influenza. These infections are very common in the community and more than 50% of the adult population experience ARI symptoms during a 6-month period and one-fifth of them will consult a general practitioner (GP) (7). As much as 90% of patients diagnosed with acute otitis media, acute sinusitis, or acute tonsillitis are treated with antibiotics in some countries (8, 9). In US, the antibiotic prescribing rate for acute bronchitis is about 70% (10), and in Australia (for GP registrars) about 73% (11), despite evidence suggesting that the antibiotic prescribing rate for this should be near 0 (12).

The solution seems at first to be straightforward: targeting the use of antibiotics in the community – which is where the greatest tonnage is prescribed and the efficacy of their use is most limited (12–15). Within about a year, over 90% of commensal antibiotic resistance dissipates (4). Despite the existence of guidelines, which recommend appropriate prescribing of antibiotics in the community, antibiotics are still prescribed for ARIs much more than guidelines recommend (16, 17). The cause of this cognitive dissonance is almost certainly more than simply a matter of prescriber personal habit (18) and requires an understanding of the various reasons, which can drive clinicians to over-prescribe antibiotics for ARIs. The aim of this paper is to provide an overview of the opportunities and strategies for reducing antibiotic use for ARIs in primary care and outline potential areas for future research.

## Strategies

**Figure 1:**
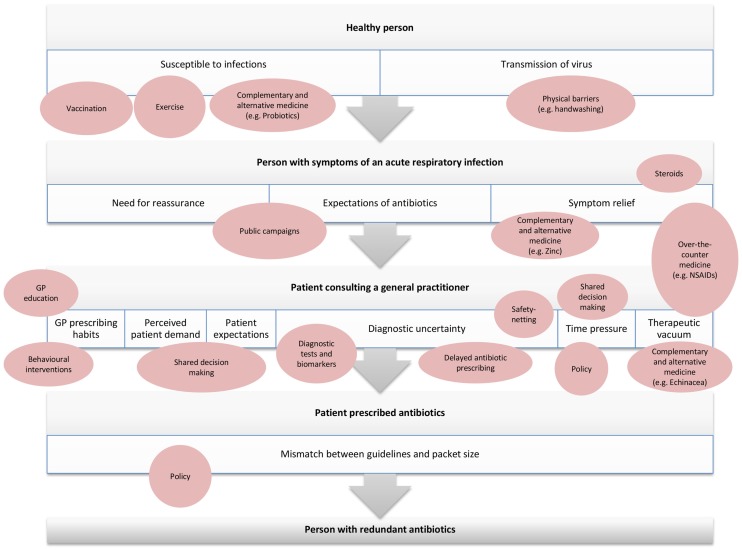
**Overview of strategies to minimize antibiotic use in primary care at each stage of the path from healthy person to antibiotic prescription for acute respiratory infection**.

Understanding the events of decision points surrounding an antibiotic prescription for ARIs in primary care can inform strategies to address the problem at each of these points. This paper considers the path from a healthy person, to a person with symptoms of ARIs, to a person consulting a GP, who may or may not be prescribed an antibiotic (Figure 1). The author team discussed and came to consensus on strategies that were likely to be used at each stage of this pathway. We then used keywords and subject headings for each strategy to search CENTRAL and PubMed (up to October 2014) to identify the highest level of evidence available for each strategy, according to conventional hierarchy of evidence (19).

## Healthy People

Of course, prevention of ARIs in the first place will reduce the whole downstream use of antibiotics.

### Vaccination

*Haemophilus influenzae* type b (Hib) is an important cause of pneumonia and meningitis in children, and the Hib vaccine is safe and effective in reducing Hib disease (20). Pneumococcal polysaccharide vaccines are effective in preventing the rarer outcome of invasive pneumococcal disease, but evidence from meta-analysis does not support the routine use of pneumococcal polysaccharide vaccines to prevent all-cause pneumonia or mortality (21). Vaccination is also often proposed for prevention of influenza. In healthy adults, it has been demonstrated to decrease the risk of symptoms of influenza and time off work, but it does not reduce complications or transmission in healthy adults (22). The etiological organisms for ARIs are numerous and the current vaccination options are unlikely to be able to influence more than a tiny proportion of infections.

### Exercise

Exercise might also reduce the incidence of ARIs, as well as their duration and severity (23).

However, these results need confirmation in other studies before exercise can be recommended for preventing ARIs, a Cochrane review is currently addressing this question (24).

### Complementary and alternative medicine

Probiotics, Echinacea, and vitamin C are sometimes suggested for preventing ARIs. Vitamin C has been used for more than 80 years, although a Cochrane review concludes that it does not reduce the incidence of common cold (25). Another Cochrane review found that trials investigating Echinacea for preventing common cold did not show statistically significant reductions in illness occurrence. However, nearly all prevention trials pointed in the direction of small preventive effects (26). Importantly, the available Echinacea products differ greatly in content of active components and due to significant differences in the preparations tested, it is not possible to draw strong conclusions about the effect of Echinacea in either preventing or treating common colds (26). Probiotics (live microorganisms) are also proposed for preventing ARIs and are found to be marginally better than placebo in terms of the number of participants who experience at least one episode of an acute upper respiratory tract infection [odds ratio (OR) 0.58; 95% confidence interval (CI) 0.36–0.92] (27). Further trials are needed to strengthen the evidence about the potential for probiotics.

### Hygiene and physical barriers

One way to diminish the number of people with ARIs is through reducing the person-to-person transmission of pathogens by applying physical barriers, hand hygiene, and so on. Respiratory virus spread can be reduced by handwashing, especially around younger children (28). It remains unclear if adding virucidals or antiseptics to normal handwashing with soap is more effective than regular soap (28). There are only limited data on the use of masks and respirators in the community to reduce transmission of virus, but their effectiveness is possibly linked to early, consistent, and correct usage (29).

## People with Symptoms of an Acute Respiratory Infection

### Need for reassurance and expectations of antibiotics

Patients with ARIs are mostly seeking information and reassurance from their GP (30, 31). However, many patients overestimate the effectiveness of antibiotics: approximately one-third of patients believe that antibiotics are effective against cold and “flu” (32), and nearly two-thirds believe that acute bronchitis requires antibiotic treatment (33). The important corollary is that those who expect an antibiotic for their ARI are nearly twice as likely to consult their GP when affected by one (7). Pharmacists or other health care providers, coming into contact with patients prior to GP consultation, may be able to change patients’ beliefs about the need for antibiotics for ARI by providing advice about self-care and information about the expected duration of the illness (7).

Public campaigns may also have a role to play in addressing public misconceptions about the effectiveness of antibiotics. There have been numerous campaigns in high-income countries to promote appropriate antibiotic use, which vary from simple, low-cost internet campaigns to expensive mass-media campaigns. Although evidence for a cause–effect relation is lacking and the effects are variable, the results of several campaigns suggest that they can decrease the inappropriate use of antibiotics. As an example, in France and Belgium, antibiotic use declined by as much as 27 and 36%, respectively (34).

### Symptom relief

Antibiotics are seldom necessary for treatment of ARIs as most of them are self-limiting conditions often caused by a virus. However, patients with ARIs feel unwell and consequently seek symptom relief (7, 31). Several symptomatic drugs as well as complementary and alternative treatments have been proposed. It is known that drugs used for symptomatic treatment of ARIs increases as antibiotic use decreases (35) and it is reasonable to assume that effective symptomatic treatment would reduce pressure on the decision to use antibiotics. Over-the-counter medicine like paracetamol (acetaminophen) and non-steroidal anti-inflammatory drugs (NSAIDs) are commonly used for symptom relief. Paracetamol may help relieve nasal obstruction and rhinorrhea in patients with common cold, but does not appear to improve other cold symptoms like sore throat, malaise, or cough (36). Another Cochrane review found that NSAIDs may improve many analgesia-related symptoms like headache, ear pain, and muscle and joint pain caused by the common cold (37). However, the results of the studies included in the review are diverse and the number of studies for one result is very small.

Steroids are also used for relieving symptoms, especially of acute rhinosinusitis. Intranasal corticosteroids have been found to have a modest effect in the resolution or improvement of symptoms of acute sinusitis [risk ratio (RR) 1.11; 95% CI 1.04–1.18] in a Cochrane review (38). Another review have demonstrated that oral corticosteroids as a monotherapy appear to be ineffective for treatment of adult patients with clinically diagnosed acute sinusitis (39), but in combination with antibiotics it may be modestly beneficial for short-term relief of symptoms in acute sinusitis [number needed to treat (NNT) = 7 for resolution or symptom improvement] (39). However, the evidence is limited, as almost all trials included in the review are performed in secondary care and there is a significant risk of bias. A large primary care trial is needed to establish whether oral corticosteroids offer additional benefits over antibiotics in clinically diagnosed acute sinusitis.

A large range of complementary and alternative medicine is available for symptomatic treatment of ARIs. Vitamin C has been proposed for treating respiratory tract infections and weak evidence suggests regular supplementation of vitamin C might be effective in reducing the duration of common cold by 8% (4–12%) in adults and 14% (7–21%) in children (25). Echinacea is also commonly used for the treatment of ARIs, although Echinacea products have not been shown to provide benefits for treating common colds (26). Probiotics have no effect on the mean duration (MD) of an episode of acute upper respiratory tract infection (MD −0.29; 95% CI −3.71 to 3.13), but their use reduces the antibiotic prescription rate (OR 0.67; 95% CI 0.45–0.98) (27).

Zinc inhibits replication of virus and has been tested in trials for the treatment of the common cold. A Cochrane review demonstrated that when zinc is administered within 24 h of onset of symptoms, it reduces the duration (days) of common cold symptoms in healthy people (mean difference of −1.03 days; 95% CI −1.72 to −0.34) (40). However, due to heterogeneity of the data results from the review should be interpreted with caution. Heated, humidified air has long been used by people with a common cold, on a theoretical basis that steam may help congested mucus drain better and heat may destroy the cold virus. A Cochrane review of six trials found that steam inhalation provided no consistent benefit in the treatment of the common cold. Three trials found benefits of steam for symptom relief with the common cold (OR 0.31; 95% CI 0.16–0.60). The sample size, however, was small and studies showed significant heterogeneity (41).

Caffeine as an analgesic adjuvant has been discussed for many years. In a Cochrane review based on 19 studies with a total of 7238 patients with different pain conditions, caffeine enhanced the efficacy of paracetamol, ibuprofen, or aspirin with a NNT of about 15 (42). However, only one trial related to ARIs were included in this review (43, 44).

Although the body of existing evidence is limited, it suggests that there is minimal benefit for the use of these non-antibiotic alternatives and larger, high quality, primary care-based studies are needed to further explore these alternatives and to develop and test new non-antibiotic treatments for ARIs.

## Patient Consulting a General Practitioner

### GP prescribing habits

There are wide variations in GPs’ antibiotic prescribing rates (45, 46) and GPs’ attitudes are a major influencing factor (18). GP education is a common component of most trials designed to address this issue (47, 48). One approach focuses on better communication skills: GPs who receive specific communication skills training prescribe fewer antibiotics for patients with ARIs [27% compared with 54% in the no training group (*P* < 0.01)] (49), even at long-term follow-up (50). A significant long-term effect has also been demonstrated on the prescribing behavior of GPs after participating in a medical educational program (between-group difference after 30-month 1.99%, 95% CI 0.56–3.42) (35). However, the effect of these comprehensive interventions seems quite modest when estimating the precise number of antibiotic prescriptions saved. The number of antibiotic prescriptions for lower respiratory tract infections was reduced from 2 prescriptions/week/GP to 1 prescription/week/GP in the study by Cals et al. (49) and a reduction in total antibiotic prescriptions from 11 prescriptions/week/GP to 8.5 prescriptions/week/GP was demonstrated in the French study (35). Although it seems very difficult to change GPs’ behavior, a simple intervention was recently carried out in US. This was a behavioral “nudge” in the format of a public commitment device – a poster-sized letter signed by clinicians and posted in the examination room. The intervention resulted in a 20% absolute reduction in inappropriate antibiotic prescribing for ARIs (51).

### Perceived patient demand and patient expectations

General practitioners often feel patients with ARIs are consulting with an expectation of antibiotics (52, 53). Back in 1998, Butler et al. found that: “Doctors knew of the evidence for marginal effectiveness, yet often prescribed for good relationships with patients” (54). This paradox is still current and may be one of the main contributors to the ongoing over-prescribing of antibiotics for ARIs. However, GPs often mistakenly assume their patients’ expectations (55). Patient satisfaction is associated with reassurance and information and not just with an antibiotic prescription (30, 31). Shared decision making may have an important role to play in addressing patient expectations and concerns. In the shared decision making process, evidence is brought into the discussion with patients, and their concerns and expectations are explicitly sought (56). The process enables clinician and patient to participate jointly in making a health decision, having discussed the options and their benefits and harms, and having considered the patient’s values, preferences, and circumstances (57). As the benefit-harm balance of antibiotics is very finely balanced, communicating this to patients may reduce their desire for antibiotics. Shared decision making has been shown to reduce antibiotic prescribing for ARIs in a number of trials (58–60), and a Cochrane review of this is currently underway (61).

### Diagnostic uncertainty

The relationship between diagnostic uncertainty and antibiotic misuse has been demonstrated in several studies (18). Clinicians typically only have a few minutes to decide if the patient has a serious infection, or is at risk of complications. The point-of-care test, C-reactive protein (CRP), has been proposed as a solution to this clinical dilemma. It is widely used in some European primary care settings (62, 63), while in other countries it is barely used (64). CRP-testing has been shown to significantly reduce antibiotic prescribing for patients with ARIs (RR 0.75, 95% CI = 0.67–0.83) (65) and it might be a useful strategy to increase patient satisfaction without compromising patient recovery (49).

In nearly 30 years, the rapid antigen detection test has been used for detection of Lancefield group A β-hemolytic streptococci (GABHS) in patients with a sore throat. Not all patients with a positive test and throat symptoms have an infection caused by GABHS (66, 67), and they may not benefit from antibiotic treatment. Still its use has been shown to significantly reduce antibiotic prescription for sore throat by more than 20% (68, 69). However, compared with the use of a clinical score alone to guide antibiotic prescribing, there is no effect of additional use of rapid antigen detection test in patients with a sore throat either for symptom management or for antibiotic use (69).

Procalcitonin is a promising biomarker for identification of bacterial infections. So far, meta-analyses have mainly investigated the use of procalcitonin as a diagnostic marker for sepsis (70, 71), but a Cochrane review from 2012 concluded that procalcitonin is a safe and effective tool to guide clinical decisions for antibiotic initiation and duration of treatment in patients with ARIs (72). However, evidence for the use of procalcitonin in primary care for ARIs is still limited and further trials are needed to assess the diagnostic accuracy of this biomarker in primary care.

General practitioners may prescribe antibiotics to prevent complications like quinsy, mastoiditis, and pneumonia, although the rates of serious complications are low in modern developed countries (73, 74). History, examination, and scores to predict bacterial infection cannot usefully identify those who will develop complications (75). GPs will need to rely on strategies such as “safety-netting” in managing the diagnostic uncertainty. The concept of “safety-netting” – that is, establishing clearly defined actions to take if the course of the illness deviates from the expected – was introduced in a book about primary care consulting in 1990s (76). Evaluation of this strategy and its effects on patient care is needed (77). Delayed prescribing is actually one form of safety-netting and is an effective strategy to deal with the diagnostic uncertainty. A Cochrane review demonstrated that delayed prescribing resulted in 32% of patients using antibiotics compared to 93% of patients in the immediate prescription group (78). Some of the included studies in the review found that in patients with acute otitis media and sore throat prescribing immediate antibiotics was more effective than delayed prescribing for fever, pain, and malaise (78). However, a newly published randomized controlled trial showed little difference in symptom control between strategies involving no prescription, immediate prescription, or delayed prescription (79).

### Time pressure

Busy GPs are more liberal with antibiotics for ARIs than less busy GPs, and use a greater proportion of broad-spectrum antibiotics (80). Unfortunately, GPs often have limited time for consultations dealing with patients with acute illness such as ARIs. In many countries, GPs are, among other things, increasingly required to take care of patients with chronic diseases. However, consultations for acute illness should also be prioritized as the pressure of limited time in primary care is correlated with antibiotic prescribing (18). Moreover, the cycle is perpetuating as patients who have been previously prescribed antibiotics for ARIs are more likely to re-consult their GP rather than self-manage for subsequent infections (81). This suggests that investing just a few minutes in shared decision making might be effective at reducing antibiotic prescribing (58) – as well as reducing future visits.

### Therapeutic vacuum

How can GPs manage the therapeutic vacuums left by not prescribing antibiotics? Over-the-counter drugs, complementary and alternative medicines, or steroids might be recommended for patients with some ARIs. Others might benefit from advice on preventing future ARIs by routine handwashing. Most patients are satisfied with a good explanation of their symptoms (30), and information of the minimal benefits of antibiotics for ARIs to understand the benefit-harm trade-off.

### Mismatch between guidelines and packet size

Often patients end up with leftover antibiotics because there is a mismatch between guidelines and the packet size of antibiotics. This is a problem because leftovers are saved and used for self-medication (82). Antibiotics that have been prescribed for one disease are subsequently used as self-medication for repeated episodes of the same disease, and also for other symptoms that are not necessarily caused by a bacterial illness (83). Redundant antibiotics are a potentially remediable source of antibiotic overuse and national regulatory authorities should ensure that the recommended dose of an antibiotic match the packet prescribed.

## Conclusion

There are numerous opportunities available in primary care for alleviating the crisis of increasing antibiotic resistance. None of these strategies or interventions on their own will greatly improve the use of antibiotics for ARIs. However, used in concert, combinations are likely to enable clinicians and health care systems to implement the strategies that will reduce antimicrobial resistance in the future.

## Conflict of Interest Statement

The authors declare that the research was conducted in the absence of any commercial or financial relationships that could be construed as a potential conflict of interest.
